# In Situ Spectroscopy of Calcium Fluoride Anchored Metal–Organic Framework Thin Films during Gas Sorption

**DOI:** 10.1002/anie.202006347

**Published:** 2020-07-15

**Authors:** Laurens D. B. Mandemaker, Miguel Rivera‐Torrente, Robert Geitner, Carolien M. Vis, Bert M. Weckhuysen

**Affiliations:** ^1^ Inorganic Chemistry and Catalysis Group Debye Institute for Nanomaterials Science Utrecht University Universiteitsweg 99 3584 CG Utrecht The Netherlands; ^2^ Organic Chemistry and Catalysis Group Debye Institute for Nanomaterials Science Utrecht University Universiteitsweg 99 3584 CG Utrecht The Netherlands

**Keywords:** calcium fluoride, gas sorption, IR spectroscopy, metal–organic frameworks

## Abstract

Surface‐mounted metal–organic frameworks (SURMOFs) show promising behavior for a manifold of applications. As MOF thin films are often unsuitable for conventional characterization techniques, understanding their advantageous properties over their bulk counterparts presents a great analytical challenge. In this work, we demonstrate that MOFs can be grown on calcium fluoride (CaF_2_) windows after proper functionalization. As CaF_2_ is optically (in the IR and UV/Vis range of the spectrum) transparent, this makes it possible to study SURMOFs using conventional spectroscopic tools typically used during catalysis or gas sorption. Hence, we have measured HKUST‐1 during the adsorption of CO and NO. We show that no copper oxide impurities are observed and also confirm that SURMOFs grown by a layer‐by‐layer (LbL) approach possess Cu^+^ species in paddlewheel confirmation, but 1.9 times less than in bulk HKUST‐1. The developed methodology paves the way for studying the interaction of any adsorbed gases with thin films, not limited to MOFs, low temperatures, or these specific probe molecules, pushing the boundaries of our current understanding of functional porous materials.

## Graphical abstract

**Calcium fluoride windows** were functionalized to facilitate the growth of surface‐mounted metal–organic frameworks (SURMOF). The resulting HKUST‐1 SURMOF was measured by in situ IR spectroscopy during CO and NO adsorption. This work showcases the practicality for utilizing such calcium fluoride windows for in‐depth in situ studies of various functional porous materials.

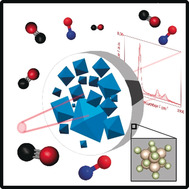

## Introduction

Metal–organic frameworks (MOFs) are known for their high porosity, tunability of components, and the resulting versatility in different potential applications. A large number of MOF structures have been reported to be active as (opto)electronic devices, catalysts, and drug‐delivery systems.^1^, ^2^, ^3^ In addition to those, the most obvious targeted applications for MOFs is as gas sensors, converters, or filters due to their high porosity and molecular sieving properties. An interesting and essential addition towards these applications is making the MOFs as (thin) films (or membranes), which allows for downgauging the material used and very precise control of the morphological properties.^4^, ^5^, ^6^ This can be done by anchoring the MOF nuclei onto a substrate, or self‐assembled monolayer (SAM) of functional organics, forming surface‐mounted MOFs (SURMOFs). Additional advantages are gained when utilizing thin films over bulk materials; anchoring the MOF creates more control over parameters, such as crystal orientation, morphology and layer thickness.^7^ However, as MOFs are synthesized as thin films, conventional characterization techniques, such as porosimetry, bulk‐based spectroscopy and (thermal) degradation techniques, for example, thermogravimetry analysis (TGA), become almost unpractical as the amount of material is very small and it is often anchored on another substrate. Nevertheless, there are new analytical techniques, like photoinduced force microscopy (PiFM), atomic force microscopy (AFM) Raman, time‐of‐flight secondary ion mass spectrometry (ToF‐SIMS,) and infrared reflection absorption spectroscopy (IRRAS), to investigate the adsorption of probe molecules on thin films, thereby bridging the materials gap.^8^, ^9^, ^10^, ^11^, ^12^

**Figure 1:**
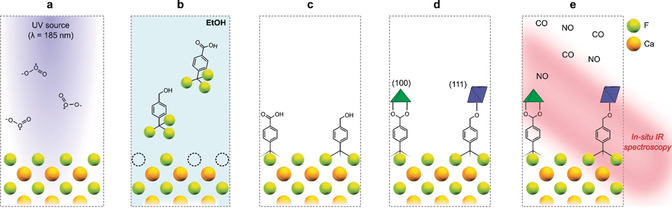
Schematic representation of the different steps to anchor a MOF material on CaF_2_ windows. a) CaF_2_ windows are pretreated by exposing them to an UV‐ozone treatment, in which the UV source creates fluoride defects on the surface. b) Trifluoromethyl‐terminated compounds are adsorbed on the defects in an ethanolic solution, c) forming a self‐assembled monolayer (SAM) on which the MOF can be anchored due to the terminating ‐COOH or ‐OH groups. d) A HKUST‐1 material is grown on the functionalized windows. Depending on the terminating group used for the SAM, the MOF (truncated) octahedral crystals will anchor in either (100) or (111) orientation. e) The optically transparent windows are then utilized for transmission FTIR spectroscopy, studying the adsorption of CO or NO, and the resulting changes in the MOF framework in situ at low temperature and increasing pressures.

In this work, as schematically shown in Figure 1 a–c, we present a practical and universally applicable method to functionalize calcium fluoride (CaF_2_) substrates with self‐assembled monolayers (SAMs), making them suitable substrates to anchor MOFs, as illustrated in Figure 1 d. As CaF_2_ is completely UV/Vis and IR transparent, this allows the grown SURMOFs to be used in a transmission FTIR spectroscopy setup, and may be applicable to any MOF topology. SURMOFs synthesized on transparent substrates have been used before in studies for which transmission experiments were necessary, as demonstrated, for example, by the work of Wöll et al., in which measurements of circular dichroism were performed on quartz‐anchored SURMOFs.^13^ In this work, we showcase a benchmark MOF structure, that is, the well‐known copper(II) 1,3,5‐benzenetricarboxylate with the topology of HKUST‐1, synthesized on CaF_2_ windows (which have a bigger transparent IR range than, for example, quartz) and studied in situ during the adsorption of CO and NO, as shown in Figure 1 e. Using this preparation technique, we were able to directly observe the structural changes inside a SURMOF material during CO and NO adsorption.

## Results and Discussion

### Calcium Fluoride Anchored Metal–Organic Frameworks

CaF_2_ is known for its insolubility in organic solvents and water, as well as for its structural stability.^14^ In order to form SAMs on any substrate, the organic compound should either form a covalent bond with the solid surface, or be strongly adsorbed by electrostatic, van der Waals, or π interactions (≈1–50 kJ mol^−1^). A plausible approach to allow for interaction with a SAM is to create surface defects. As is illustrated in Figure 1 a, fluoride defects were formed by treating CaF_2_ with an UV‐ozone source (185 nm).^15^, ^16^, ^17^, ^18^ After the creation of surface vacancies, the CaF_2_ windows were put into a concentrated solution of fluorine‐bearing aromatics to grow a SAM as illustrated in Figure 1 b. We have tried to use both fluoride (F)‐ and trifluoromethyl (CF_3_)‐terminated molecules. Confirming the presence of a single layer and the type of coordination can be a practical challenge, as monolayers of organics are often too thin to measure using spectroscopic techniques. Hence an experimental approach was used based on the anchored orientation of HKUST‐1. It is well‐known that HKUST‐1 films can be grown in different orientations. When the SAM is terminated by ‐COOH groups, the film is grown along the paddlewheel secondary building units in the (100) orientation; when it is terminated by ‐OH groups, the film is grown along the axial sites in the (111) orientation, as is illustrated in Figure 1 d.^19^, ^20^ By growing HKUST‐1 on the functionalized CaF_2_ windows we could determine whether the utilized SAM indeed displays anchoring behavior in the rationally predicted orientation. If not, it would not be possible to grow HKUST‐1 at all, or if a layer is formed of these organics but not a SAM with the proper functional groups pointing out of the surface, the resulting MOF might be anchored but will not have the desired orientation and/or will be heterogeneously distributed.

**Figure 2:**
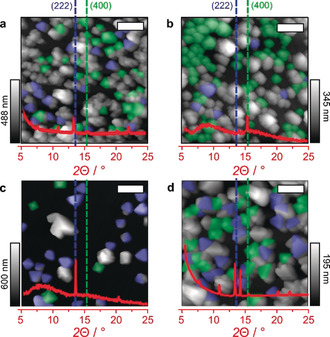
AFM micrographs and the corresponding XRD patterns of a HKUST‐1 material synthesized using 100 cycles of the LbL approach on CaF_2_ windows functionalized with a) FBCOOH, b) TFMBCOOH, c) TFMBOH, and d) TFMBCOOH, but without the UV‐ozone pretreatment. The selective ordering displayed in (b) and (c) highlights the necessity for a trifluoromethyl group in the potential self‐assembled monolayer (SAM). The fact that (d) shows no ordering can be noted in contrast to (b), illustrating that the UV‐ozone pretreatment is a requirement to form the SAM. The white scale bars are 1 μm.

Three types of SAM‐forming organics were studied: 4‐(trifluoromethyl)benzoic acid (TFMBCOOH), 4‐(trifluoromethyl)benzyl alcohol (TFMBOH), and 4‐fluorobenzoic acid (FBCOOH). After the UV‐ozone treated CaF_2_ windows were left in SAM solution for ≈17 h, HKUST‐1 SURMOF was grown in a layer‐by‐layer (LbL) fashion as reported by Delen et al.^21^ Figure 2 displays the atomic force microscopy (AFM) images and X‐ray diffraction (XRD) patterns of the formed HKUST‐1 films. An AFM micrograph and XRD diffractogram of pure CaF_2_ can be found in Figure S1 for comparison, showing a clean surface. When FBCOOH was utilized as SAM, anchored HKUST‐1 was found, and both truncated octahedral (100) and octahedral (111) features can be seen in Figure 2 a. Therefore, this aromatic does not form a SAM in which the ‐COOH groups are free to bind the HKUST‐1. In contrast, having the same carboxylic acid group, but a trifluoromethyl instead of a fluoro group, the TFMBCOOH SAM displays preferential formation of the (100) morphology as seen in Figure 2 b, meaning that the trifluoromethyl group must adsorb onto the surface, while the carboxylic acid groups are open to anchor the MOF. This is further confirmed with the TFMBOH SAM, which leads to a uniform formation of the (111) features as is seen in the AFM image and XRD pattern as seen in Figure 2 c.

The stronger adsorption of trifluoromethyl groups is expected as 1) the three fluorides have slightly more electron density than a single fluoride: quantum‐chemically calculated Voronoi deformation density (VDD) of −0.083, −0.085, and −0.09 for TFMBCOOH and −0.093, −0.097, and −0.086 for TFMBOH, compared to −0.07 for FBCOOH, as suggested by the calculated electron density surfaces (Figure S2), and more importantly 2) the tetrahedron‐shaped ‐CF_3_ groups can “encapsulate” the open Ca^2+^ sites. Density functional theory (DFT) was used to show that the interaction of the CF_3_‐terminated molecule with a Ca^2+^ ion resulted in a lower Gibbs free energy (Δ*G*_CF3‐Ca_=−218.7 kJ mol^−1^) than for its F‐terminated counterpart (Δ*G*_F‐Ca_=−209.1 kJ mol^−1^). More information and discussion about this theoretical approach can be found in the Supporting Information (SI, Section S2). As a control experiment, a pristine CaF_2_ window was treated using the same functionalizing steps with TFMBCOOH, but without the UV‐ozone pretreatment, which resulted in both (100) and (111) coordination as becomes clear from Figure 2 d. This highlights the necessity to create fluoride surface defects using UV‐ozone, as otherwise, the fluoro‐terminated compounds are not able to properly interact with the surface. Synthesizing a HKUST‐1 material on both pristine and UV‐ozone treated CaF_2_ windows without any SAM results in roughly no growth at all, as becomes clear from Figure S3.

**Figure 3:**
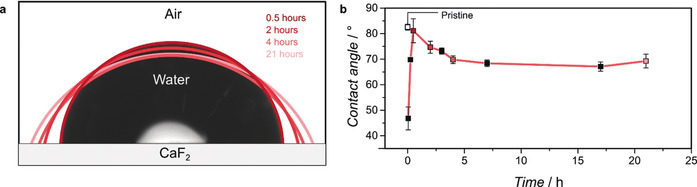
Contact angle measurements, performed by applying water droplets (a) on CaF_2_ that had been functionalized with TFMBCOOH for increasing periods. As the anchoring molecules increase the hydrophilicity of the surface due to the terminating ‐COOH groups, the contact angle between water and the surface decreases. After ≈5 h (b) the contact angle is stable, meaning the self‐assembled monolayer (SAM) is formed. The different red points in (b) represent the droplets shown in (a). The error bars represent the standard deviation (*σ*) for the three measurements performed.

An essential part of growing oriented MOFs is the quality of the anchoring monolayer. When the UV‐ozone treated CaF_2_ surface is not submerged into the SAM solution long enough, the substrate will only be partially functionalized resulting in poor film growth. To study the actual formation of a SAM versus exposure time, contact angle measurements were performed, as represented in Figure 3 a, using a water droplet on the CaF_2_ surface (which was exposed to TFMBCOOH in ethanol) for different durations. The results are shown in Figure 3 b. Here, the red points correspond to the droplets found in Figure 3 a and the error bars represent the standard deviations (*σ*) on three different measurements. The pristine CaF_2_ window is hydrophilic (Figure 3 b, white point), having contact angles around 83°. After UV‐ozone treatment, the hydrophilicity of the sample increases drastically, resulting in a contact angle of approximately 40°. We believe this is due to the removal of organic surface contaminations, accompanied by the formation of defects on which water can be adsorbed.

However, when the surface is exposed to an ethanolic solution of TFMBCOOH for only 0.5 h, the hydrophilicity decreases again, resulting in a contact angle similar to the pristine substrate (81°). As the time of exposure to the TFMBCOOH solution is increased, the contact angle decreases until after ≈5 h, when it stabilizes around 70°. As the amount of ‐COOH terminated molecules on the surface increases with increasing time, the surface becomes more hydrophilic. Due to the stabilization of the contact angle and the resulting mono‐orientation of HKUST‐1, we assume that after ≈5 h the SAM is formed. To check the stability of the SAM, we measured the substrate functionalized for 17 h (67°, *σ*=1.8°), and again after it was stored for 12 days (Figure S4). The contact angle increased to 74° with an unusually large error (*σ*=7.8°) compared to when freshly functionalized, indicating partial loss of the SAM. As a result, all MOF samples in this work were synthesized directly after surface functionalization.

### FTIR Spectroscopy with CO and NO as Probes

**Figure 4:**
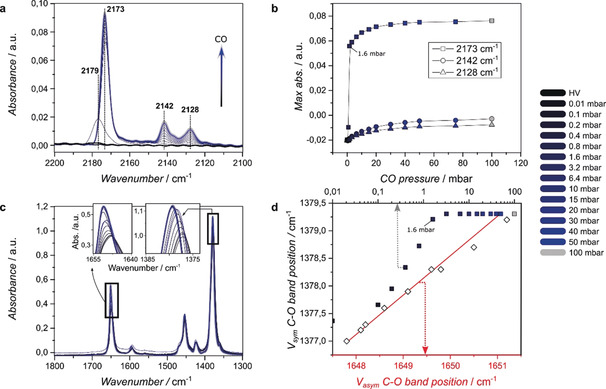
In situ FTIR spectroscopy results obtained during the a) adsorption of CO on a HKUST‐1 material grown on functionalized CaF_2_ with increasing pressure (0–100 mbar). b) Three major vibrational bands are increasing in their intensity, representing CO being adsorbed on the different sites within the HKUST‐1 material. The C‐O bands corresponding to the metal–linker interaction (c) are also influenced, as they blue‐shift with increasing CO pressure. Both *v*_asym_ and *v*_sym_ are correlated (d, red) at all pressures, which reveals that the coupling is not influenced. Additionally, the shift (correlated to the stretch, d, gray) stops after 1.6 mbar, highlighting the correlation with the adsorption of CO on the Cu^2+^ species.

With the developed SURMOF preparation technique and the related CaF_2_‐anchored MOFs in hand, we turned our attention to the study of the gas‐sorption properties of the HKUST‐1 materials. There has been a debate on the adsorption of gases on the different sites of HKUST‐1, especially on the adsorption on the Cu sites. To address this ongoing debate in the literature, we have used in situ IR spectroscopy on our synthesized HKUST‐1 SURMOF to study the mechanism during the adsorption of two probe molecules, CO and NO. The big advantage of using a thin‐film material in probe FTIR spectroscopy is the low saturation and minimal overlap of vibrational bands, as has been highlighted by Wöll et al.^22^ Additionally, because we apply the IR‐transparent CaF_2_ window as the substrate in a transmission approach, we avoid reflecting or refracting/scattering effects, obtaining very well‐defined vibrational bands, which theoretically allow for the direct application of the Lambert–Beer law. Materials‐wise, our HKUST‐1 materials were synthesized in an identical manner as described before, only now with 800 layers, which yields very defect‐poor HKUST‐1 (vide infra) with features having a median thickness of 650 nm.^21^ Even after so many synthesis cycles, the resulting SURMOF is mostly mono‐oriented due to the coordination on the TFMBCOOH SAM, as is shown in Figure S5. Figure 4 a shows the adsorption of a mixture of 10 % CO/He on HKUST‐1 with increasing pressures (starting from high vacuum 10^−5^ and up to 100 mbar) in the FTIR spectra. As stated, the observed peaks are clearly separated and there is no background interference. Interestingly, we observe the formation of three major peaks. The peak observed at 2179–2173 cm^−1^ is ascribed to the Cu^2+^⋅⋅⋅CO adduct.^22^, ^23^ As can be noted in Figure 4 b, this peak increases rapidly in intensity at low CO pressures (<1.6 mbar). The peak shift (2179 to 2173 cm^−1^) shows that relatively high coverage of CO is reached at low CO pressures,^23^ which is expected for thin films. The second peak at 2142 cm^−1^ is not always reported in the literature, as it is often lost in the more intense surrounding peaks. However, Bordiga et al. observed the formation of this band specifically at higher coverages and ascribed it to “liquid‐like” state CO bands, which are similar to CO in the condensed phase.^23^, ^24^ The peak at 2128 cm^−1^ is ascribed to Cu^+^⋅⋅⋅CO adducts. The origin of the Cu^+^ is yet unclear: whilst some groups claim it is due to Cu_2_O impurities originating from either the Cu acetate precursors or thermal reduction of the Cu^2+^ paddlewheel in the activation steps prior to catalysis or sorption,^22^, ^23^, ^25^, ^26^, ^27^ other groups state that it originates from defective Cu^+^/Cu^2+^ dimeric paddlewheel species, which are reversible redox sites.^28^, ^29^ Recent work from Wöll et al. even utilizes this redox behavior to catalyze the CO oxidation at low temperature using the interplay between the Cu^2+^ and Cu^+^ paddlewheel species.^30^ Furthermore, as de Vos et al. state that the Cu_2_O impurities can be observed visibly, we are certain that at least larger impurities are not the origin of Cu^+^ as we do not observe any unexpected morphological features on the nanoscale (Figure 2).^25^ Besides, as has been pointed out by Wöll et al., the conditions we applied during the synthesis at room temperature, and the activation before the CO probe FTIR spectroscopy at 100 °C for 2 h, are not likely to induce the formation of large Cu_2_O impurities.^22^, ^23^ Additionally, the XRD diffractogram in Figure S5b shows no indication that Cu_2_O species are present after the gas‐sorption, which would have reflections at 22, 27, and 32° (Cu_2_O) or 19 and 28° (CuO).^11^ One can argue that the small amount of species would have a negligible signal next to the MOF, but note that we are looking at SURMOFs where the CaF_2_ is still visible using AFM (Figure 2), a top‐down technique, demonstrating the low volume of MOF present; hence the small copper oxide nanoparticles would take up a significant amount of our mass. The C‐O antisymmetrical (*v_asym_*) and symmetrical (*v_sym_*) stretching vibrations representing metal–linker interactions (1645 cm^−1^ and 1380 cm^−1^, respectively) show no abundant sign of uncoordinated paddlewheels as seen in Figure 4 c.^21^ We conclusively assume then that the presence of the 2128 cm^−1^ band is indeed linked to the natural amount of Cu^+^ paddlewheel species.^31^

As the thin film does not adsorb too extensively due to the low amount of material, the MOF fingerprint area was not saturated, and we could track the metal–linker interaction of the paddlewheel versus CO pressure (Figure 4 c). First of all, we highlight that the shift observed for the *v_asym_* is perfectly correlated to the *v_sym_* shift, as plotted in Figure 4 d (red line). This means that the coupling effect on the C‐O vibrations of the linker due to the coordination with the metal is not influenced by the CO pressure. When we consider the shift of the *v_sym_* as a function of CO pressure (gray points) we note a huge increase up until 1.6 mbar. Then, the metal–linker bands do not shift any further. Hence, this shift is correlated to the CO adsorbed on the Cu^2+^ sites shown in Figure 4 b (2173 cm^−1^). The CO adsorbing on the Cu^2+^ is donating electron density to the metal via σ donation, which in turn weakens the bonds (Cu–O) with the coordinated oxygens from the ‐COO group from the linker. As a result, the C−O bonds in the COO group have a higher energy, leading to the observed blue‐shift. This means that at relative low coverages, the CO is bound strongly on the coordinately unsaturated sites of the Cu^2+^ in the paddlewheel. In parallel, as becomes clear from Figure 4 d the CO is also adsorbed on the Cu^+^ sites and into its “liquid‐like” state.

**Figure 5:**
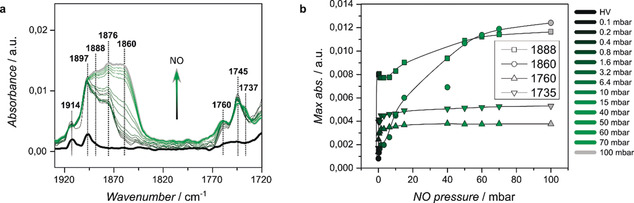
In situ FTIR spectroscopy results obtained during the a) adsorption of NO on a HKUST‐1 material grown on functionalized CaF_2_ with increasing pressures (0–100 mbar) at 85 K. The N‐O stretching bands are formed after the initial introduction of NO. When the pressure is further increased, several bands even increase further in intensity or are formed. b) As the NO binds strongly to Cu^2+^ species and eventually also on Cu^+^, the intensity of such bands is plotted versus NO pressure.

Whilst CO tends to bind preferably to Cu^+^ species, NO is known to favor adsorption on Cu^2+^ species. Figure 5 shows the adsorption of 10 % NO/He on HKUST‐1 (the same sample as used for the CO adsorption before), with increasing pressures (10^−5−^100 mbar) at 85 K. Many (shoulder) bands are formed when NO is introduced into the MOF, which do not increase further as a function of pressure, for example, the aromatic backbone vibrations (1914 and 1897 cm^−1^).^32^ The 1888 cm^−1^ vibration is ascribed to mono‐nitrosyls, or Cu^2+^⋅⋅⋅NO adducts,^23^, ^28^, ^32^, ^33^ whilst the band at 1876 cm^−1^ is attributed to divalent Cu‐nitrosyls in slightly different coordination environments, which indicates structural defects,^28^ and that at 1860 cm^−1^ is reported to represent Cu^+^⋅⋅⋅(NO)_2_ dimers formed solely at low temperatures.^32^ At lower wavenumbers, Cu^+^ nitrosyl vibrations found are explained by extra‐framework species (1737 cm^−1^) or *trans* isomers of Cu^+^⋅⋅⋅(NO)_2_ adducts (1770–1760 cm^−1^). The absorbance of a selection of vibrational bands has been plotted versus pressure in Figure 5 b. The C‐O vibrations of the linkers coordinated to the metal do not shift after the initial introduction of NO, as is shown in Figure S6. This is expected, as NO molecules do not back‐donate electron density to the Cu species as CO molecules do.^28^ From the plotted absorbance maxima it becomes clear that NO is first adsorbed on paddlewheel Cu^2+^ species, increasing the intensity of the 1888 cm^−1^ band. In contrast, the intensities of the paddlewheel Cu^+^⋅⋅⋅NO adducts only increase at higher NO pressures after the 1888 cm^−1^ band increased. This behavior is completely unrelated to the increase in defective extra‐framework Cu^+^ species, which seemingly follows the typical Langmuir adsorption model. The band intensity from the Cu^+^⋅⋅⋅NO adducts and extra‐framework species (the 1770‐1730 cm^−1^ region), relative to the intensities found for the Cu^2+^‐related bands (the 1900–1830 cm^−1^ region), remains low (A_Cu+⋅⋅⋅NO_ 1760 cm^−1^/A_Cu_^2^_+⋅⋅⋅NO_ 1888 cm≈0.5) compared to results reported for identical measurements on the powder bulk HKUST‐1 (A_Cu+⋅⋅⋅NO_ 1760 cm^−1^/A_Cu_^2^_+⋅⋅⋅NO_ 1888 cm≈2) at similar pressures of NO.^32^ This confirms the observations made on the CO adsorption: the amount of extra‐framework Cu and/or (other) defects is low, and so the SURMOF consists of a significant amount of paddlewheel‐coordinated Cu species dominating the adsorption of probe gases, confirming the findings from Daturi et al.^28^

**Figure 6:**
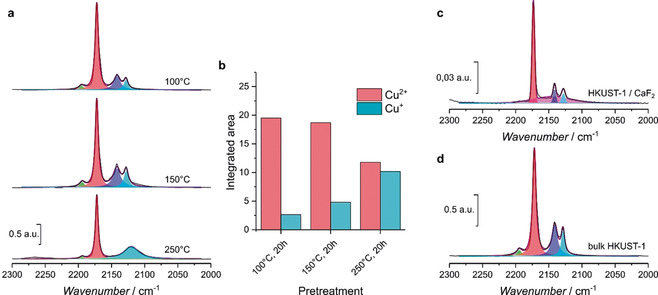
FTIR spectra (black), their deconvoluted component bands (green, red, blue, and aqua) and the reconstructed spectra (purple) obtained after dosing ≈100 mbar 10 % CO/He on HKUST‐1 a) pretreated for 20 h at 100 °C (top), 150 °C (middle), and 250 °C (bottom) under vacuum. b) Integrated areas of the deconvoluted components at ≈2170 cm^−1^ (red) and ≈2120 cm^−1^ (aqua) for the different pretreatment temperatures representing the Cu^2+^⋅⋅⋅CO and Cu^+^⋅⋅⋅CO adducts, respectively. Similar spectra and deconvoluted component bands for c) HKUST‐1 SURMOF anchored on CaF_2_, used in Figure 4, and bulk HKUST‐1 pretreated at 100 °C.

Quantifying the exact amount of Cu^+^/Cu^2+^ species is a challenge, as the strong redox behavior and dependency on conditions, pretreatments, and the influence of the measurement itself might shift the ratio, as is substantiated by the highlighted debate on the nature of these species. In the case of IR spectroscopy, this results in the lack of individual (integrated) molecular adsorption coefficients for the specific bands observed in this work, although such coefficients have been found in the past on MOFs containing different metals and Cu‐based zeolites and metal oxides.^34^, ^35^ Nevertheless, we feel it would be inaccurate to assume these coefficients to be similar for MOFs, as their dynamic structure and chemical composition are undoubtedly different from these reported compounds; hence we cannot quantitatively compare the amount of Cu species in the film with earlier reported HKUST‐1 materials. As an approximation of the ratio of Cu^+^/Cu^2+^, Szanyi et al. deconvoluted their spectra and worked with the integrated area of the different bands.^28^ Applying a similar approach, as can be seen in Figure 6, we, first of all, prove that the pretreatment of the MOF strongly influences the resulting Cu^+^/Cu^2+^ ratio, also in our measurement conditions. Figure 6 a displays the bands for bulk HKUST‐1 pretreated at 100, 150, and 250 °C for 20 h, after dosing ≈100 mbar of 10 % CO/He. The higher temperature during the pretreatment of the MOF (in vacuum) results in decreasing amounts of Cu^2+^ and increasing amounts of Cu^+^ (see also Table 1). The higher temperature results in the reduction of the Cu^2+^ paddlewheel species, presumably due to oxidative decarboxylation as reported by Wöll et al.^30^ Arguably the pretreatment at 250 °C also induces decomposed impurities, likely copper oxides, as the Cu^+^⋅⋅⋅CO band around 2120 cm^−1^ becomes very broad.^27^ To minimalize differences that the pretreatment of the MOF might induce on the IR bands resulting from CO adsorption, we compare our SURMOF to bulk which were both pretreated at 100 °C.

**Table 1:** Integrated areas of the deconvoluted Cu^2+^⋅⋅⋅CO and Cu^+^⋅⋅⋅CO bands shown in Figure 6, representing the different amounts of Cu^2+^ and Cu^+^.

Sample	Pretreatment	Cu^2+^⋅⋅⋅CO [cm^−1^]	Cu^+^⋅⋅⋅CO [cm^−1^]	Cu^+^/Cu^2+^ band ratio
bulk HKUST‐1	100 °C, 20 h	19.51	2.66	0.136
bulk HKUST‐1	150 °C, 20 h	18.68	4.81	0.258
bulk HKUST‐1	250 °C, 20 h	11.77	10.16	0.86
HKUST‐1 SURMOF/CaF_2_	100 °C, 2 h	0.46	0.056	0.12
bulk HKUST‐1	100 °C, 5 h	16.12	3.66	0.23

Their resulting spectra, after dosing with ≈100 mbar of 10 % CO/He, are plotted in Figure 6 c,d. Evident from Figure 6, we used another band in the deconvolution of the SURMOF spectrum. Using only the four distinct bands as implied before by Szanyi et al. resulted in a poor fit and an excessive integrated area for the Cu^+^⋅⋅⋅CO adduct for this specific sample.^28^ To tackle this issue, we have performed a blank test on 50 layers HKUST‐1/CaF_2_ as can be seen in Figure S7, which reveals the presence of gaseous CO adsorbed on the surface, showing the roto‐vibrational bands, from 2200–2100 cm^−1^_._ For bulk experiments, these bands are negligible as the absorption intensities of the Cu‐adsorbed CO and “liquid‐like” CO in the pores are orders of magnitude higher. For the blank experiment, these bands are not visible as the MOF was too thin and could not be detected using our setup. Nevertheless, the broad band from 2200–2100 cm^−1^ is present, revealing it to be unrelated to the amount of MOF. As the SURMOF HKUST‐1 used for the gas sorption experiments (800 layers) is still only <1.0 μm thick, resulting in relatively low adsorption maxima overall, we therefore take this broad gas band into consideration for the deconvolution (pink deconvoluted band, Figure 6 c) resulting in a more accurate fit. Clearly, the CaF_2_‐mounted HKUST‐1 SURMOF contains a distinct lower amount of Cu^+^ species than the bulk. The integrated areas and resulting Cu^+^/Cu^2+^ ratios are represented in Table 1.

As a direct division of the ratios (Cu^+^_bulk_/Cu^2+^_bulk_)/(Cu^+^_SURMOF_/Cu^2+^_SURMOF_) completely disregards the molecular adsorption coefficients (as they are identical in both ratios, their actual value is irrelevant), we quantitatively report the defect‐poor nature of HKUST‐1 films, having 1.9 times less Cu^+^ species than their bulk counterpart. Furthermore, we observe the lack of the 2192 cm^−1^ band (green deconvoluted) band, which was tentatively assigned by Bordiga et al. to the *v_sym_* mode of Cu^2+^⋅⋅⋅(CO)_2_ complexes.^26^ Even though this follows the expected trend for Cu^2+^ species as a function of pretreatment temperature in Figure 6 a, we disregard it for the integrated area calculations done on the bands from Figure 6 c,d as we argue that this weak band was not strong enough to be observed in the SURMOF HKUST‐1 due to the low amount of material.

## Conclusion

We have shown that CaF_2_ windows can be functionalized with a self‐assembled monolayer (SAM) to allow for the growth of MOF thin films, which can then be easily studied with conventional FTIR spectroscopy, even in situ or during operando conditions. It was found that inducing defects on the CaF_2_ surface is essential before forming the SAM. Additionally, the organic species used should contain a ‐CF_3_ group to bind to the surface. By terminating the molecule with either ‐OH and ‐COOH groups, we were able to show the preferential ordering of the resulting HKUST‐1. We also showed that >5 h was needed to form the SAM in ethanolic solution. Using this methodology, we were able to measure CO and NO probe FTIR spectroscopy for increasing pressures at low temperatures on MOF thin films using a conventional spectroscopic setup. We observed that CO is strongly adsorbed on the paddlewheel Cu^2+^ species in the MOF, which was correlated to the shift in spectral bands from the MOF linker itself. CO was also adsorbed on Cu^+^ species, but the amount of extra‐framework Cu was found to be low. The adsorption of NO highlighted the presence of paddlewheel Cu^+^ species and confirmed that the amount of defects was low. Therefore, we conclude that SURMOFs synthesized using the layer‐by‐layer (LbL) synthesis method are defect‐poor, but still do possess Cu^+^ species in their paddlewheel conformation. By directly comparing the SURMOF to its bulk counterpart, we find the Cu^+^/Cu^2+^ ratio to be 1.9 times lower for the thin film. In general, CaF_2_ functionalization presents an opportunity to involve SURMOFs in a quantitative characterization, and the methodology presented here thus allows for further studies of MOFs or other thin‐film functional porous materials, as their adsorbing active sites or intermediates can be directly compared to each other or their bulk counterparts using in situ spectroscopic tools. This creates opportunities to study SURMOF (thin) films in many processes for which a spectroscopic toolbox can be utilized, like catalytic conversions, probe molecule adsorption, or sensor‐related studies, like for example, the behavior of HKUST‐1 as water detector.^36^ Additionally, this approach could be extended to (and used to compare) other synthesis techniques,^4^ like one‐pot solvothermal self‐assembly,^8^ spin‐coating,^11^, ^37^ and the emerging chemical vapor or atomic layer deposition methods.^38^, ^39^, ^40^, ^41^

## Conflict of interest

The authors declare no conflict of interest.

## Supporting information

As a service to our authors and readers, this journal provides supporting information supplied by the authors. Such materials are peer reviewed and may be re‐organized for online delivery, but are not copy‐edited or typeset. Technical support issues arising from supporting information (other than missing files) should be addressed to the authors.

Supplementary
