# Supplementary material for: In Situ Spectroscopy of Calcium Fluoride Anchored Metal–Organic Framework Thin Films during Gas Sorption
Source: Angew Chem Int Ed Engl. 2020 Jul 15;59(44):19545–52. doi: 10.1002/anie.202006347 (PMC7689770; doi:10.1002/anie.202006347)
Supplement: Supplementary file 1 — Supplementary [file ANIE-59-19545-s001.pdf]

Angewandte Chemie
Eine Zeitschrift der Gesellschaft Deutscher Chemiker

Supporting Information

In Situ Spectroscopy of Calcium Fluoride Anchored Metal–Organic Framework Thin Films during Gas Sorption

*Laurens D. B. Mandemaker, Miguel Rivera-Torrente, Robert Geitner, Carolien M. Vis, and Bert M. Weckhuysen**

anie_202006347_sm_miscellaneous_information.pdf

S1. Additional Figures

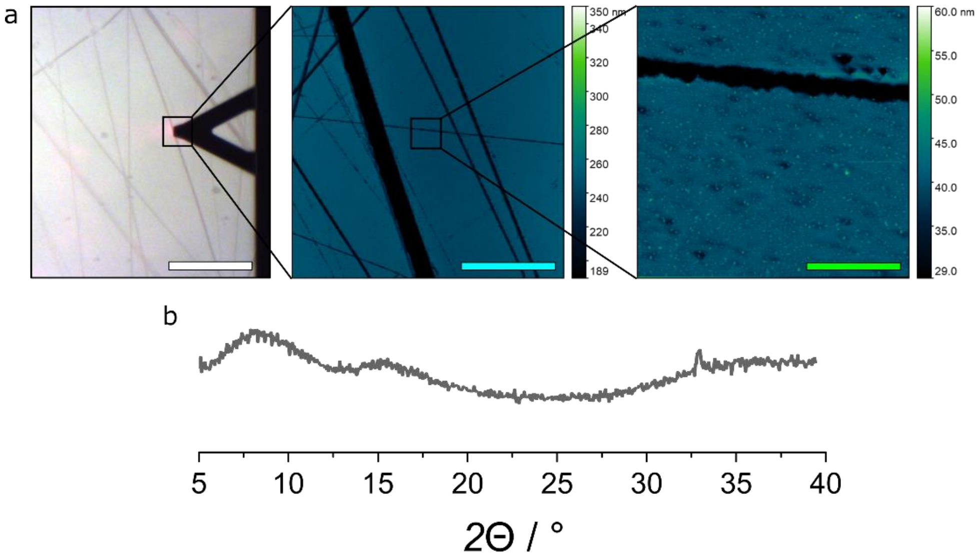

Figure S1. a) Optical images and atomic force microscopy (AFM) images of a pristine calcium fluoride (CaF_2) window. The material shows larger (macro) and smaller (micro) trenches, and a small surface roughness (RMS = 1.22 nm) on the planes in between. White scale bar = 100 µm, cyan scale bar = 10 µm, green scale bar 1 µm. b) X-ray diffraction (XRD) pattern of the pristine CaF_2, measured in the same range as the XRD patterns in the main manuscript, showing that CaF_2 possesses no dominant reflections there (only a minor around 33°).

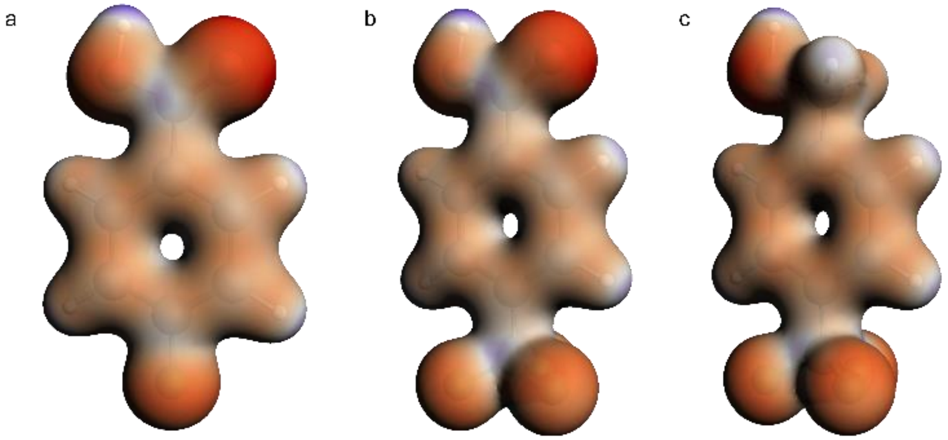

Figure S2. Electron density maps of the organic anchoring molecules, a) FBCOOH, b) TFMBCOOH and c) TFMBOH, showing relative electrostatic potential for each atom. The Voronoi deformation density (VDD) for the FBCOOH fluoride is slightly lower (-0.07) than for the TFMBCOOH (-0.083, -0.085 and -0.09) and TFMBOH (-0.093, -0.097 and -0.086) fluorides.

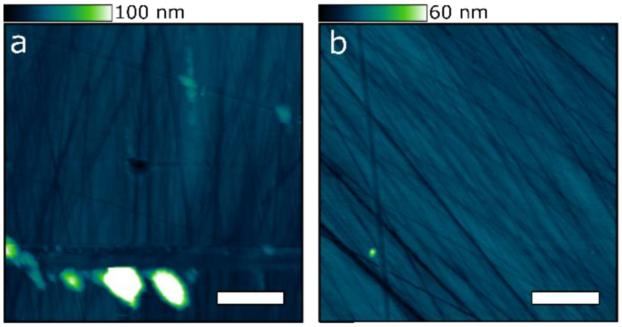

Figure S3. Atomic force microscopy (AFM) micrographs of 100 cycles of HKUST-1 synthesized in layer-by-layer (LbL) fashion on a) pristine CaF_2 and b) UV-ozone treated CaF_2, demonstrating that without proper functionalization with an anchoring self-assembled monolayer (SAM), HKUST-1 is not anchored on these windows.

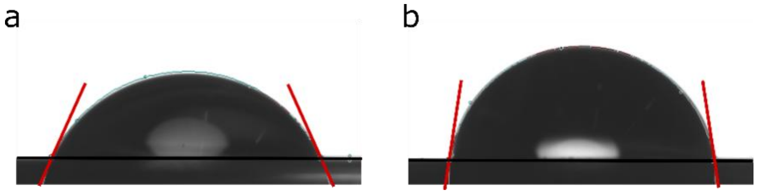

Figure S4. Contact angle measurements on CaF_2 windows, which have been pretreated by UV-ozone and functionalized with self-assembled monolayers (SAM) for 17 h. The angle was measured between the window and a water droplet (a) directly after formation of the SAM and (b) after the same sample was stored for 2 weeks. The contact angle is significantly increased, from 67° (σ = 1.8°) to 74° with an unusual large error (σ = 7.8°), showing the SAM to degrade over these storage durations.

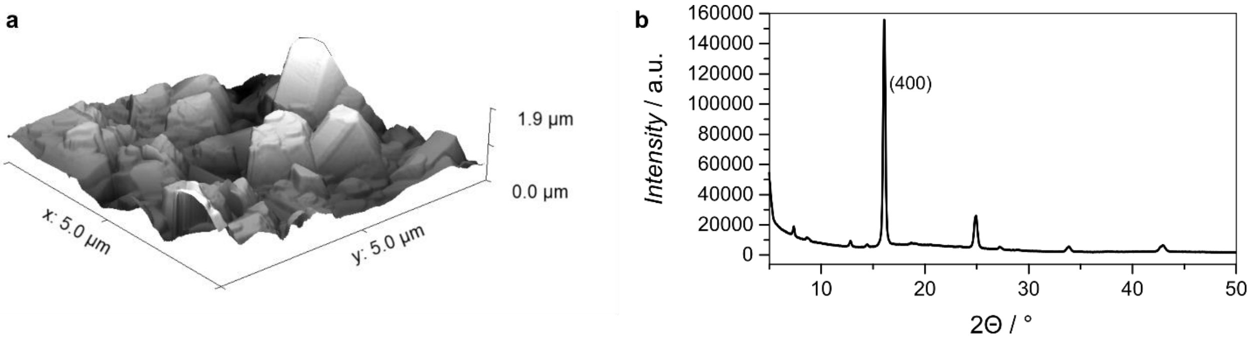

Figure S5. a) Atomic force microscopy (AFM) micrograph of HKUST-1 on TFMBCOOH/CaF_2, 800 layers, showing a dense closed layer of HKUST-1 features with a median height of 650 nm. b) Corresponding X-ray diffraction (XRD) pattern of the sample, highlighting the strong (100) orientation from the utilized -COOH terminated self-assembled monolayer (SAM), as is not obvious anymore from the AFM micrograph due to the large amount of material.

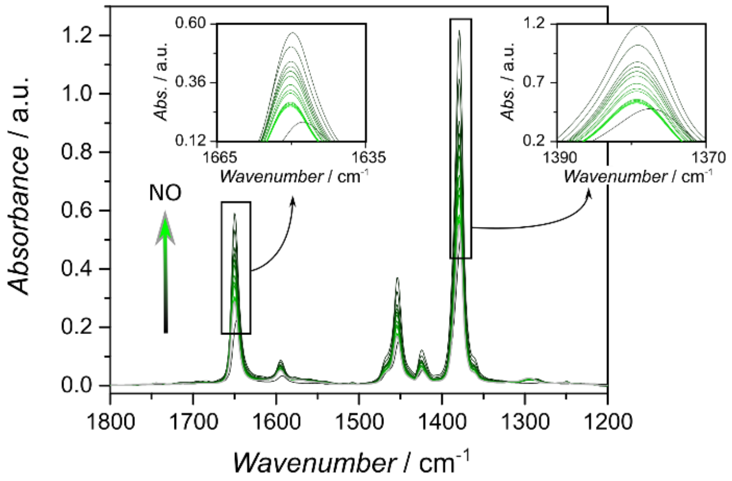

Figure S6. FT-IR spectra recorded during the adsorption of NO (10^{-5}-100 mbar) on HKUST-1. The ν_{asym} and ν_{sym} of the linkers C-O stretch are decreasing in intensity as the NO pressure increases. Nevertheless, the position of the bands remains identical (after the initial exposure).

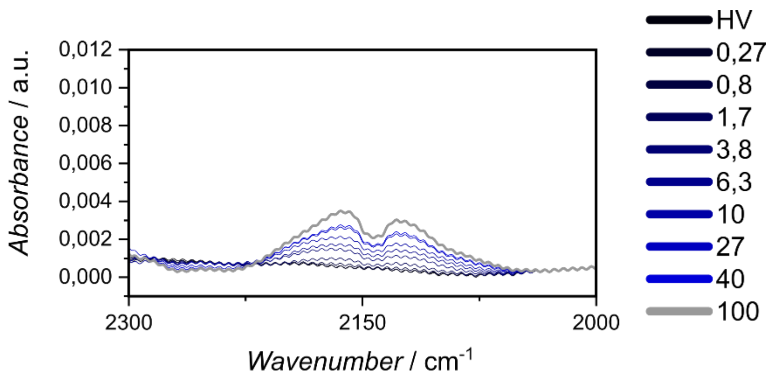

Figure S7. FT-IR spectra recorded during the adsorption of CO (10^{-5}-100 mbar) on 50 layers of HKUST-1/CaF_2. The broad, split band represents the rotovibrational CO being adsorbed on a surface. Note that the intensity is similar to the deconvoluted band (pink) in Figure 6c in the main manuscript.

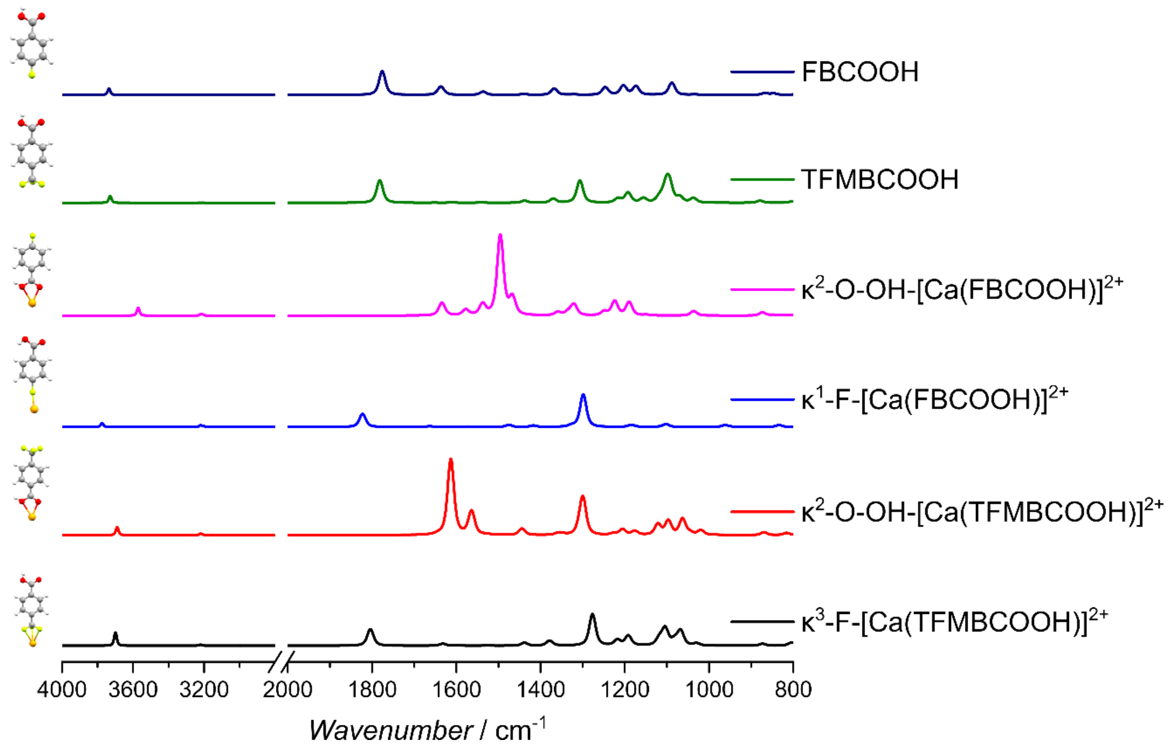

Figure S8. Generated FT-IR spectra for FBCOOH and TFMBCOOH molecules, either free or interacting (with their respective -F or -O groups) with a single Ca^{2+} ion.

S2. Discussion on Density Functional Theory Calculations

Density functional theory (DFT) calculations were performed as an estimation on the binding energy of the different organic terminated groups (-COOH, -F, -CF_3) on a single Ca^{2+} ion *in vacuo*, as we believe the organics to bind on the Ca^{2+} sites in the CaF_2. To keep the calculations within a reasonable time frame we focussed on just the interaction between the different fluoriated organics and one Ca^{2+} ion. We are aware of the fact that surface effects, fluorine ions, solvent molecules and the large number of neighboring ions, will also influence the calculated binding energy between the Ca^{2+} site and the organic molecules. These simplifications are the same for all Ca^{2+}-organics pairs and can thus be considered a systematic error. Nevertheless, the different energies can still be compared with each other as all calculations feature the aforementioned simplifications. As discussed in the main text our calculations show that the coordination of a Ca^{2+} ion towards -CF_3 groups is stronger than to -F groups. This is in line with our experimental observations form the SURMOF formation. This confirms that -CF_3 groups are needed to properly form the SAM and to subsequently anchor the MOF in a controlled way.

S3. Experimental Details

a. Materials Synthesis

4-(Trifluoromethyl)benzoic acid (98%, Sigma-Aldrich), 4-(trifluoromethyl)benzyl alcohol (98%, Sigma-Aldrich) or 4-fluorobenzoic acid (98%, Sigma-Aldrich) were dissolved (20 mM) in ethanol (abs, VWR Chemicals) and stored in a fridge. IR-polished calcium fluoride (13 mm * 0.5 mm, supplied by Crystran Ltd, Poole) windows were first put in an UV-ozone cleaner (175 nm) 30 min per side. Then they were directly put in a glass vial vertically agains the cylindrical wall with 2 mL of SAM solution (described above) and put at 45°, to ensure both sides of the window were accessible. After 17h (for standard synthesis) the windows were washed with ethanol and dried using compressed air.

The HKUST-1 was synthesized using a layer-by-layer approach with a SILAR automated dipping robot. For one cycle, the windows were dipped for 2 min in subsequently M $Cu(NO_3)_2$ (99+%, Sigma-Aldrich) ethanolic or trimesic acid (95%, Sigma-Aldrich) ethanolic solution. In between, they were washed at 350 rpm for 60 s in pure ethanol. The samples used for AFM and XRD consisted of 100 cycles, the sample used for CO-FT-IR spectroscopy consisted of 800 cycles. Bulk HKUST-1, or copper benzene-1,3,5-tricarboxylate, was obtained from Sigma-Aldrich (Basolite® C300).

b. Materials Characterization

Atomic force microscopy (AFM) was performed using a Bruker Multimode 8 and ScanAsyst-Air SiN_3 cantilevers (F = 0.4 N/m) in non-contact mode, except for the 800 layers HKUST-1 sample which was measured with a HA_NC cantilever with monocrystal silicon tip (F= 12 N/m) in tapping mode. The data was post-treated using Gwyddion, and open-source SPM software.[1] The images were flattened by substracting a background plane and alligning the rows using a “median-of-differences” function. The green and blue color mask, representing the different coordination

shapes in Figure 2, were drawn manually using photoshop, to highlight the specific features. The median height of the sample with 800 cycles was determined by setting a region corresponding to CaF_2 in Figure S5 to z = 0, and then the median height was taken from Gwyddion's "Statistical Quantities" function.

X-ray diffraction (XRD) was measured using a Bruker Bruker-AXS *D2* Phaser powder X-ray diffractometer in Bragg-Brentano geometry, using Co $K_{\alpha 1,2}$ = 1.79026 Å, operated at 30 kV. The measurements were carried out between 5 and 50° using a step size of 0.05° and a scan speed of 1 s, with a 0.1 mm slit for the source. Simulated XRD patterns were obtained by processing the corresponding *.cif* files with VESTA® (λ = 1.79026 Å, FWHM = 0.2).

FT-IR spectroscopy was performed on a PerkinElmer System 2000 instrument (64 scans, 4 cm^{-1} resolution, MCT detector cooled with liquid N_2, cell with KBr windows). The films deposited on the CaF_2 were mounted on a stainless-steel cell and activated under the conditions at $p < 10^{-5}$ mbar (temperatures) described in the main text. For the bulk HKUST-1, 10 mg was pressed into a self-supporting pallet and then mounted in similar fashion and activated as indicated in the main manuscript. Thereafter, the cell was cooled with liquid N_2 temperature down to 85 K, and a mixture of 10% CO/He v/v (Linde AG, 99.999%) or 10% NO/He v/v (Linde AG, 99.9%) was dosed stepwise into the cell via a stainless-steel manifold and a valve to the pressures indicated in the main text. Deconvolutions of the spectra were performed using Origin 2017, utilizing the "Multiple peak" fit function on the 2300 – 2000 cm^{-1} range fitting Lorentz curves. The deconvoluted peaks were then integrated over the full 2300 – 2000 cm^{-1} range to obtain the utilized integrated areas reported in Table 1 in the main manuscript.

Contact angle measurements were performed dropping UV and milipore-filtered demineralized water on the different substrates. Images were recorded using a Dataphysics OCA 15 optical contact angle measuring setup equipped with a single direct dosing module (SD-DM) and the resulting contact angles were calcultated using SCA20 software.

c. Quantum Chemical Calculations

To model the chemical interaction between TFMBCOOH respectively FBCOOH and the CaF_2 surface at a reasonable computational demand only a single Ca^{2+} ion was used to simulate the surface. As discussed earlier this drastic simplification is useful to get the binding energies between Ca^{2+} and the fluorinated organics.

All quantum chemical simulations were performed using DFT with the global hybrid functional B3LYP[2] and a non-augmented double-ζ-basis set of slater-type orbitals with one polarization function (DZP)[3] implemented in the Amsterdam Density Functional (ADF) Modeling Suite 2016.[4] No electron approximations were made (no frozen core), relativistic effects were included via the scalar zeroth-order regular approximated (ZORA) relativistic equation and the numerical quality was 'Good'.

The relaxed equilibrium structures of TFMBCOOH, FBCOOH, κ^2-O-OH-[Ca(TFMBCOOH)]$^{2+}$, κ^2-O-OH-[Ca(FBCOOH)]$^{2+}$, κ^3-F-[Ca(TFMBCOOH)]$^{2+}$ and κ^1-F-[Ca(FBCOOH)]$^{2+}$, have been obtained, while subsequent vibrational analyses determined minima of the 3N-6 dimensional potential energy (hyper-)surfaces as well as vibrational frequencies and infrared intensities (Figure

S8). For all Ca^{2+} coordination compounds the C-F bond distances were locked to the distance in the non-coordinating molecules.

S4. Additional References

[1] Nečas, D.; Klapetek, P. Gwyddion: An Open-Source Software for SPM Data Analysis. *Open Phys.* **2012**, *10*, 181-188.

[2] a) C. Lee, W. Yang, R. G. Parr, *Phys. Rev. B* **1988**, *37*, 785–789; b) A. D. Becke, *J. Chem. Phys.* **1993**, *98*, 5648–5652.

[3] a) C. Fonseca Guerra, J. G. Snijders, G. te Velde, E. J. Baerends, *Theor. Chem. Acc.* **1998**, *99*, 391–403; b) G. te Velde, F. M. Bickelhaupt, E. J. Baerends, C. Fonseca Guerra, S. J. A. van Gisbergen, J. G. Snijders, T. Ziegler, J. *Comput. Chem.* **2001**, *22*, 931–967; c) E. van Lenthe, E. J. Baerends, *J. Comput. Chem.* **2003**, *24*, 1142–1156.

[4] E. J. Baerends, T. Ziegler, A. J. Atkins, J. Autschbach, D. Bashford, O. Baseggio, A. Bérces, F. M. Bickelhaupt, C. Bo, P. M. Boerritger et al., ADF2016, SCM, Theoretical Chemistry, Vrije Universiteit, Amsterdam, The Netherlands, https://www.scm.com, 2016.
